# Regional and national guideline recommendations for digital ano-rectal examination as a means for anal cancer screening in HIV positive men who have sex with men: a systematic review

**DOI:** 10.1186/1471-2407-14-557

**Published:** 2014-08-01

**Authors:** Jason J Ong, Marcus Chen, Andrew E Grulich, Christopher K Fairley

**Affiliations:** Melbourne School of Population and Global Health, University of Melbourne, 580 Swanston Street, Carlton, Victoria 3053 Australia; Melbourne Sexual Health Centre, 580 Swanston Street, Carlton, Victoria 3053 Australia; Central Clinical School, Monash University, Clayton, Victoria 3168 Australia; Kirby Institute, University of New South Wales, Sydney, NSW 2052 Australia

**Keywords:** Anal cancer screening, Systematic review, Guidelines, Digital ano-rectal examination, HIV positive, Men who have sex with men

## Abstract

**Background:**

Although anal cancer is common in HIV positive men who have sex with men, few centres offer systematic screening. Regular digital ano-rectal examination (DARE) is a type of screening that has been recommended by some experts. How widely this forms part of HIV management guidelines is unclear.

**Methods:**

The protocol was registered prospectively (CRD42013005188; http://www.crd.york.ac.uk/PROSPERO/). We systematically reviewed 121 regional and national HIV guidelines and searched for guidelines from http://hivinsite.ucsf.edu/global?page=cr-00-04#SauguidelineX, PubMed and Web of Science databases up to 5^th^ August 2013 for recommendations of DARE as a means of anal cancer screening in HIV positive MSM. Guidelines were examined in detail if they were clinical guidelines, including both prevention and treatment protocols and were in English. Guidelines were excluded if they were restricted to limited areas (e.g. antiretroviral therapy only, children or pregnant women, strategies for prevention/testing). Information was extracted regarding recommendation of DARE as a screening method, the frequency of DARE recommended, target population for screening and the strength of evidence supporting this.

**Results:**

30 regional and national guidelines were included and examined in detail. Only 2 recommended DARE. The ‘European AIDS Clinical Society Guidelines’ recommends DARE every 1–3 years for HIV positive MSM whilst the ‘US Guideline for prevention and treatment of opportunistic infections in HIV-infected adults and adolescents’ recommends an annual DARE for the HIV + population in general. None of these guidelines specify the age of commencing screening. In each case, the highest level of evidence supporting these two recommendations was expert opinion.

**Conclusions:**

Few HIV guidelines discuss or recommend DARE as a means of anal cancer screening. Studies of the efficacy, acceptability and cost-effectiveness of DARE are needed to assess its role in anal cancer screening.

## Background

Anal cancer is defined as cancers arising from the squamous and glandular epithelia of the anus. The great majority are squamous cell carcinomas (SCC) [1–4]. Anal cancer has received little attention given its rarity in the general population (~1–2 in 100,000) [1–3, 5]. However, incidence is higher among men who have sex with men (MSM) especially those who are HIV-positive. A recent meta-analysis estimated the anal cancer incidence rate to be 46 per 100,000 in HIV positive MSM [6]. However there have been reports as high as 131 to 137 per 100,000 in large US cohorts of HIV-positive MSM, and a number have reported an increasing incidence in the post-highly active antiretroviral therapy (HAART) era [7, 8]. Anal cancer is now the most common non-AIDS defining cancer in HIV infected people in Australia [9].

The morbidity associated with anal cancer and its treatments is significant. Although local excision may be considered for small well differentiated anal cancers, in most instances chemo-radiotherapy is needed for treatment [10, 11] with its potential long term side effects such as impotence. The quality of life for someone with anal cancer has been estimated to be worse than those with either oropharyngeal, vaginal, vulvar or penile cancer [12].

Despite the high incidence, and morbidity of anal cancer in some populations, there is still no consensus recommendation for how to effectively screen for anal cancer for those at highest risk (i.e. HIV positive MSM). There are two approaches suggested 1) detecting early cancers using regular DARE or 2) detecting precursor lesions using an anal cytology-based program with diagnostic high resolution anoscopy (HRA) to identify high-grade squamous intraepithelial lesion (HSIL) [13], which can then be treated using a variety of ablative or other treatments (typically, DARE is also performed in this approach). Other potential approaches may include HRA alone [14] or DARE with subsequent cytology/HRA [15]. Some centres have adopted the stance that given the relatively high burden of anal cancer in the HIV population, anal-cytology based screening and treatment for HGAIN should be implemented. They argue that the similarities to the cervical cancer model justifies screening until this evidence is available [16, 17]. However there remains significant barriers to implement an anal cytological screening service including low sensitivity to detect HSIL due to a large percentage of HIV-positive MSM with abnormal cytology [6], lack of high-resolution anoscopists and no evidence from randomized controlled trials that treatment of HSIL prevents development of anal cancer [17]. At this point in time the majority of HIV clinicians do not offer an anal cytology screening service outside a limited number of centres [18, 19].

So whilst evidence of screening and treating HSIL continues to gather and clinical expertise develops, should we implement the model of a regular DARE to detect early cancer? Survival from anal cancer is markedly higher if it is treated at an early stage. For instance, the US National Cancer Institute data on 6,411 patients showed that tumours less than 2 cm at diagnosis had 80% 5 year-survival compared with 45–65% when the tumour was more than 2 cm and 20% for tumours that had metastasized [20]. A case series of 38 HIV-positive men with tumours less than 3 cm had a 5 year cancer specific survival of 85% compared to zero in those with tumours greater than 3 cm [21]. A French series of 69 patients with anal cancers less than 1 cm reported a 100% 5 year survival [17].

Currently, most anal cancers are detected when they are locally advanced with mean tumor size of 3 to 4 cm [11, 22, 23]. Thus, earlier diagnosis than currently occurs, for example through the routine use of DARE, has great potential to lead to reduced morbidity. Experts have suggested that all individuals at higher risk for anal cancer should have a regular DARE [24].

Although some published articles suggest regular DARE as a means of screening [25–27], currently only a minority of patients at highest risk for anal cancer receive a regular DARE as a part of their HIV care [28]. Our aim was to determine if this low rate of DARE was because few national guidelines recommended it, or because of a poor uptake of existing guidelines. We systematically reviewed national HIV guidelines to evaluate recommendations for the implementation of regular DARE as part of routine HIV care.

## Methods

### Search strategy

The protocol was prospectively registered in the ‘International prospective register of systematic reviews’ (http://www.crd.york.ac.uk/PROSPERO; CRD42013005188). We initially searched for major HIV guidelines through the comprehensive list found on http://hivinsite.ucsf.edu/global?page=cr-00-04#SauguidelineX (accessed 5^th^ August 2013). This website compiles the latest HIV national guidelines from around the world. We searched these 121 HIV guidelines for recommendations regarding the use of DARE for early anal cancer detection. Secondly, we searched the US National Library of Medicine’s PubMed (http://ncbi.nlm.nih.gov/pubmed) and Thomson Reuter’s ISI Web of Science (http://thomsonreuters.com/thomson-reuters-web-of-science/) databases using the following text string ‘(anal OR anus OR ano*) AND (cancer OR carcinoma OR neoplasm OR malignancy OR ‘squamous cell carcinoma’ OR ‘squamous cell cancer’) AND (screen*) to search in the field ‘title’.

### Inclusion and exclusion criteria

Guidelines were examined in detail if they were clinical guidelines, including both prevention and treatment protocols. This led to excluding 91 guidelines because they were either guidelines aimed at children or pregnant women (n = 30), specific antiretroviral protocols (15), strategies for HIV prevention/testing (12), or was not published in English (28). 6 other guidelines were excluded because they were discussing HIV infected health care workers (1), nutrition guidelines (1), duplicates (2), home-based care program (1) and work place program (1).

In searching PubMed and Web of Science, the titles and abstracts were examined and 346 full text publications were fully appraised as they met the following criteria: English language, anal cancer or its screening in the HIV positive population. 6 published articles were identified as ‘guidelines’ and one additional guideline was identified through searching the reference lists of reviewed publications. Of the 7 publications, 4 guidelines were excluded as they were not national guidelines [10, 29–31] and another 2 were identified as national guidelines but excluded as they did not mention DARE [32, 33]. As a subanalysis, we searched for original articles that utilized DARE alone as a means for anal cancer screening.

### Quality assessment and data extraction

Each of the 30 national HIV guidelines was reviewed for statements regarding anal examination and/or DARE including who to screen and how frequently to screen. The level of evidence quoted to support such a recommendation was also captured. The level of evidence was assessed using the US Preventive Services Task Force for ranking evidence for the effectiveness of screening [34].

Level I denotes evidence from at least one properly designed randomized controlled trial. Level II denotes evidence from well designed controlled trials without randomization, cohort or case-control analytic studies or multiple time series with or without the intervention. Level III denotes evidence from opinions of respected authorities, based on clinical experience, descriptive studies or reports of expert committees.

## Results

**Figure 1:**
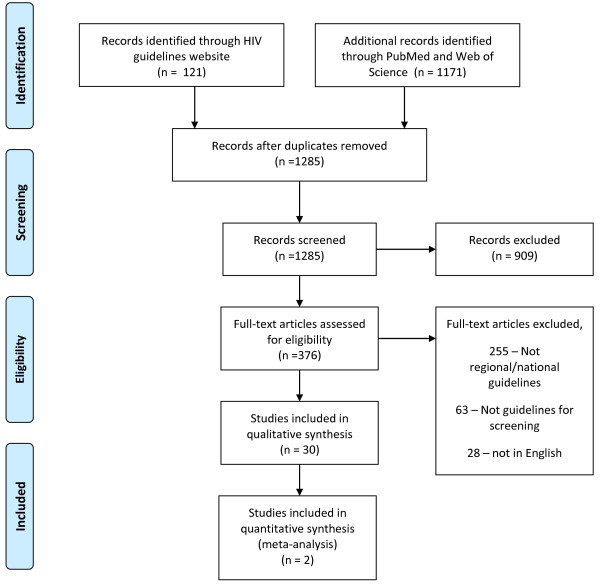
**Search Strategy.**

**Figure 2:**
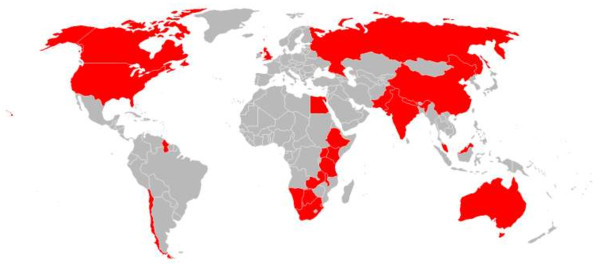
**HIV National guidelines evaluated (in red).**

Figure 1 is a flow diagram for the literature search and guideline selection. Tables 1 and Figure 2 summarizes the countries covered by the national guidelines reviewed. Full text was not accessible for the countries in bold because the guidelines were not in English.

**Table 1:** **Number of HIV guidelines reviewed**

Reviewed (number of guidelines)	Not reviewed because was not available in English
*Regional*	
World (15)	Latin America (2)
East Asia and Pacific (1)	
Eastern Europe and Central Asia (2)	
Carribean (1)	
South and South East Asia (3)	
Western Europe (3)	
*National*	
Australia (1)	Argentina (2)
Botswana (1)	Brazil (6)
Canada (5)	Bhutan (1)
China (2)	Chile (1)
Egypt (3)	Colombia (1)
Ethiopia (5)	France (1)
Guyana (1)	Germany (2)
Hong Kong (2)	Mexico (2)
India (5)	Mozambique (1)
Kenya (4)	Spain (5)
Malawi (1)	Ukraine (3)
Malaysia (2)	Uruguay (1)
Namibia (2)	
Nepal (1)	
Pakistan (2)	
Russian federation (2)	
South Africa (4)	
Swaziland (1)	
Tanzania (2)	
Uganda (2)	
United Kingdom (8)	
United States (7)	
Zambia (4)	

Two guidelines specifically discuss DARE (Table 2). The European AIDS Clinical Society Guidelines specifically recommend that ‘homosexual men’ should have a ‘digital rectal exam ± Papanicolau test’ with a screening interval of ‘1–3 years’. The evidence of benefit was quoted as ‘unknown advocated by some experts’ [35]. Although this is the most specific recommendation of all the guidelines reviewed, there was no explicit description of how this recommendation was derived nor was it referenced. These guidelines did not explicitly describe the process that was undertaken to arrive at a recommendation and there was no ‘level of evidence’ grading of this specific recommendation for anal cancer screening.

**Table 2:** **Guidelines that mention DARE as a means for anal cancer screening**

Guidelines	Recommendation	Target population	Frequency of DARE	Level of evidence
European AIDS Clinical Society Guidelines [33]	‘digital rectal exam +/- Papanicolau test’	HIV positive MSM	Every 1–3 years	III (expert opinion)
US Guideline for prevention and treatment of opportunistic infections in HIV-infected adults and adolescents [38]	‘digital rectal examination as an important procedure to detect masses on palpation that might be anal cancer’	Not specified	Annually	III

The second guideline that refers to DARE was the USA’s ‘Guideline for prevention and treatment of opportunistic infections in HIV-infected adults and adolescents’. This guideline did not recommend DARE but made the statement that it may be useful: ‘An annual digital anal examination may be useful to detect masses on palpation that could be anal cancer’. The guideline did not recommend a frequency for DARE or identify a specified population that should be screened and specifically did not limit their suggestion to HIV positive MSM. The recommended level of evidence for this is BIII, which refers to ‘moderate recommendation for the statement’ and ‘expert’ opinion. The process of derivation of this recommendation was described as through those with expertise in this area reviewing the literature to produce draft guidelines. The recommendations were then reviewed by the Opportunistic Infections Working Group of National Institutes of Health. Final versions were reviewed and endorsed by CDC, the National Institutes of Health and the HIV Medicine association of the Infectious Diseases Society of America.

We did identify two other guidelines that referred to the issue of anal cancer but did not make specific recommendations about DARE. The British HIV association guidelines for HIV-associated malignancies do not recommend DARE and implies that patients do their own anal examination [33]. The guideline stated that the ‘role of annual anal cytology and anoscopy is not yet proven; however, patients should be encouraged to check and report any lumps noticed in the anal canal’. This again was based only on expert opinion (Level III) with no references to any published studies. It is important to note that these guidelines are currently being revised but in light of the lack of published evidence for DARE, we do not believe that the recommendation is likely to alter at this stage. The World Health Organization’s Treatment and care protocols for the European Region [36] acknowledge that ‘anal cancer is strongly associated with HPV infection and it is significantly more likely among MSM who are HIV infected’ and that ‘any patient suspected of cancer should be examined by an oncologist and referred to the oncology clinic as needed’. However no guidance is provided as to what examination is needed. This again is based only on expert opinion (Level III) with no references to any published studies.

In our subanalysis of original articles utilizing DARE as a screening tool, we found one article that described the acceptability of DARE to a HIV-positive MSM population [37]. However this study did not provide any efficacy data for DARE.

## Discussion

Although DARE is recommended by experts [25–27], this has not been reflected in HIV guidelines. In our review of regional and national HIV guidelines, we found only one that recommended regular DARE and another that considered it may be useful. The highest level of evidence for this was expert opinion. This highlights the need for more data on whether DARE is effective for the early detection of anal cancer in HIV positive MSM. A recent study of 138 HIV-positive MSM with anal cancer found that early anal cancer detection was possible in asymptomatic men if they were closely followed up with regular DARE [38]. However, to date, there have not been any studies evaluating whether widespread implementation of regular DARE in those at highest risk for anal cancer (i.e. HIV-positive MSM) would reduce the morbidity and mortality from anal cancer and its management. Currently DARE is not commonly undertaken. One study found that within a HIV clinic, only 10% of their patients were receiving anal cancer screening either by DARE and/or cytology [28]. Yet anal cancer in HIV positive MSM is the most common non AIDS defining cancer in this group, and as frequent as the common cancers in the general community such as bowel cancer, for which screening programs are in place.

To our knowledge, this is the first examination of regional and national HIV guidelines to quantify the degree of support for DARE to detect early anal cancers. Our systematic review specifically did not review the literature on recommendations for DARE in the general community because the incidence of this cancer is about 100 fold higher in HIV positive MSM. This means that recommendations made in the general community may be quite different because the positive predictive value, negative predictive value and costs will also be very different. We did not evaluate guidelines that were in languages other than English. This excluded 28 of the 121 guidelines and so it is possible that important recommendations based on higher levels of evidence were missed.

The limited range of guidelines in relation to DARE reflects the absence of studies addressing the key screening issues in relation to prevention of morbidity from anal cancer [39]. Some of these criteria are clearly satisfied in relation to DARE screening for anal cancer. These are that anal cancer is an important health problem in people with HIV, it has a recognizable early stage and effective treatment leading to better outcomes for early stage diagnosis. Other criteria for an effective anal cancer screening test are not yet met. DARE has not yet been proven to be a simple and acceptable test, the distribution of test values in the target population is not clear, there is no general agreement on who should be screened and how, and the cost of the procedure is not well documented. However, recent data has provided data suggesting a high level of acceptability of the procedure and suggested minimal additional health-care cost [37]. Questions remain in relation to the impact of having a regular DARE on quality of life measures and costs associated with false negative and false positive results. Future screening studies must also include an evaluation of the potential for increased anxiety and worry [40]. Furthermore, there remains no evidence of DARE’s efficacy or efficiency (i.e. sensitivity, specificity, positive predictive value, negative predictive value), the acceptability of DARE for doctors, nor any cost-effectiveness evaluation of DARE. If DARE is to be recommended into routine HIV care, this information is urgently needed.

## Conclusion

Anal cancer is an urgent health priority for HIV-positive MSM. Although some experts have recommended regular DARE as a means of detection of anal cancer, few HIV guidelines discuss or recommend DARE as a means of anal cancer screening. There is a need for further studies of the efficacy, acceptability and cost-effectiveness of DARE before its role in anal cancer screening can be determined.

## Abbreviations

DARE: Digital ano-rectal examination
HIV: Human immunodeficiency virus
HSIL: High-grade squamous intra-epithelial lesion
MSM: Men who have sex with men
SCC: Squamous cell carincoma.
